# Selenium and cancer risk in CHEK2 mutation carriers

**DOI:** 10.1186/1897-4287-10-S3-A19

**Published:** 2012-04-20

**Authors:** Satish Gupta, Katarzyna Jaworska, Katarzyna Durda, Grzegorz Sukiennicki, Magdalena Muszyńska, Anna Jakubowska, Jan Lubinski

**Affiliations:** 1 International Hereditary Cancer Centre, Department of Genetics and Pathology, Pomeranian Medical University, Szczecin, Poland; 2 Postgraduate School of Molecular Medicine, Warsaw Medical University, Warsaw, Poland

## 

Checkpoint kinase 2 (CHEK2) is as an important signal transducer of cellular responses to DNA damage and acts as a tumour suppressor gene. Mutations in the CHEK2 gene have been shown to be associated with increased risks to several cancers. In Poland four mutations in CHEK2 gene (1100delC, IVS2+1G>A, I157T, del5395) have been identified. Studies on our population provided evidence that CHEK2 truncating mutations are associated with increased risk of cancer in the thyroid, breast and prostate. The I157T has been found to be associated with increased risk of breast, prostate, colon, kidney and thyroid cancer. The variability in penetrance and cancer expression in mutation carriers probably can be explained by influence of other genetic or environmental factors.

Our preliminary data indicate that serum selenium level is associated with breast/ovarian cancer risk in Polish BRCA1 carriers. Both genes, BRCA1 and CHEK2, belong to the same pathway, however clinical presentation of cancer phenotype in carriers of mutation in particular gene is different eg. in respect to cancer location or tumour pathological features. It may suggest presence of distinct genetic factors which modify cancer risk in BRCA1 and CHEK2 carriers.

Aim of the study was to check if Se concentration as well as alterations in genes coding selenoproteins can be a risk factors for cancer development in CHEK2 mutation carriers. In this study we will determine serum Se concentration and analyze genes coding selenoproteins in ~100 CHEK2 mutation carriers affected by different cancers (eg. breast, prostate, colon, kidney, lung, larynx) and ~100 unaffected CHEK2 carriers matched to the cancer cases.

We analysed for Se concentration and alterations of 13 selenoproteins in 58 CHEK2 carriers constituting 29 pairs, each composed of cancer patient and unaffected control matched by: CHEK2 mutation type (missense or protein truncating), sex, year of birth, smoking status (pack-years +/- 20%) and cancer family history among I° relatives.

We observed lower Se concentration in cancer patients than in controls (70.5µg/l vs. 76.8 µg/l), however due to low number of pairs this difference was not statistically significant. We found some evidence that specific genotypes in tested selenoprotein genes may be associated with different cancer risk depending on Se concentration, however this observation has also to be verified in larger number of pairs.

